# The OROCHI (Observational Research of Clinical Course After mamusHI) Study: A Prospective, Observational, Multicenter Study on the Efficacy of Mamushi (Gloydius blomhoffii) Antivenom Serum

**DOI:** 10.7759/cureus.64877

**Published:** 2024-07-18

**Authors:** Kotaro Kaneda, Tomoaki Inoue, Yasutaka Koga, Takeshi Yagi, Masaki Todani, Takashi Nakahara, Motoki Fujita, Ryosuke Tsuruta

**Affiliations:** 1 Advanced Medical Emergency and Critical Care Center, Yamaguchi University Hospital, Ube, JPN

**Keywords:** gloydius blomhoffii, japan, clinical course, snake bites, antivenom

## Abstract

Background: Evidence on the efficacy of mamushi antivenom serum is limited.

Objective: To investigate the effectiveness of mamushi (*Gloydius blomhoffii*) antivenom serum.

Methods: The Observational Research Of the Clinical course after mamusHI bite (OROCHI) study was a prospective multicenter study conducted at 24 hospitals in Japan. Patients hospitalized due to mamushi bite were registered. The primary endpoint was the length of hospital stay. Secondary endpoints were adverse effects, pain (numerical rating scale), and grade of swelling. We performed a cohort analysis to compare outcomes between patients treated with mamushi antivenom serum (antivenom group) and those who were not treated with the serum (no-antivenom group).

Results: Overall, 106 patients were registered across 18 hospitals between April 22, 2020, and October 31, 2022. Of these, 92 were eligible for the analyses, with 53 and 39 in the antivenom and no-antivenom groups. The median (interquartile) length of hospital stay was not significantly different between the antivenom and no-antivenom groups (5 (3-6) days vs. 3 (1-8) days, *P* = 0.369). In multivariable analysis, the adjusted odds ratio for a hospital stay of >4 days was 1.331 in patients treated with mamushi antivenom serum (95% confidence interval (CI) = 0.744‒2.015, *P* = 0.574) and 6.154 in patients treated with cepharanthine (95% CI = 1.442-26.258, *P* = 0.014). Pain and the grade of swelling were worse in the antivenom group than in the no-antivenom group up to 24 h after arrival, but there were no differences in these outcomes after 48 h.

Conclusion: Although the effectiveness of mamushi antivenom serum in reducing the length of hospitalization was not demonstrated, beneficial effects on pain and swelling were observed.

## Introduction

Mamushi (*Gloydius blomhoffii*) bites are the most common type of snakebite in Japan, occurring throughout the country with a higher frequency in the south and an estimated incidence of 1.67 per 100,000 people over six months from July to December [1]. Although the mortality rate is low (0.2%), it was reported that 4.6% of cases developed hypovolemic shock, 3.3% developed acute kidney injury, and 1.7% developed disseminated intravascular coagulation [1]. Therefore, mamushi bites are a life-threatening environmental hazard in Japan.

Mamushi antivenom serum is commercially available. It is produced by purifying and processing serum from horses immunized with venom or toxoids. Although mamushi antivenom serum is used to treat severe mamushi bites, some institutions have a policy against using it because there is sparse evidence regarding its effectiveness. To the best of our knowledge, only retrospective studies have examined the efficacy of mamushi antivenom serum [2-7]. Cepharanthine, an alkaloid isolated from *Stephania cepharantha* Hayata, is also thought to inactivate mamushi venom and is less harmful, but there is also little evidence for its effectiveness [8]. In this context, the multicenter, prospective Observational Research Of Clinical course after mamusHI bite (OROCHI) study was conducted to investigate the clinical course of patients with mamushi bites and the effects of various treatments. Our objective was to determine the effectiveness of mamushi antivenom serum using data from the OROCHI study. Our hypothesis was that mamushi antivenom serum reduces the length of hospital stay.

## Materials and methods

Patients

Twenty-four Japanese facilities participated in the OROCHI study; the facilities were recruited at scientific meetings and by inviting affiliated hospitals. Each facility participated in the study after obtaining approval from their institutional review board. All cases of mamushi bite admitted to the participating facilities between April 22, 2020, and October 31, 2022, were included in the study. The diagnosis of mamushi bite was made by the treating physician according to sightings, bite marks, and symptoms. Sighting included a verbal or photographic description from an eyewitness or the presence of a physical specimen. Informed consent was waived because of the non-interventional nature of the study. The study was supported by a Grant-in-Aid for Scientific Research (19K09435).

The present analysis included all cases registered in the OROCHI study, excluding cases aged <18 years, non-hospitalized cases, and cases with an interval of >24 h between the bite and hospital arrival. The exposure arm consisted of those who were treated with mamushi antivenom serum (antivenom group), while the control arm included those who were not treated with antivenom (no-antivenom group). The administration of mamushi antivenom serum, cepharanthine, and analgesics was at the discretion of the attending physician.

Data collection

The OROCHI study used the University Hospital Medical Information Network’s INDICE Cloud to collect information on patient background characteristics, findings at hospital admission, clinical course, treatment, and outcome of eligible patients. Pain was assessed using an 11-point numerical rating scale (NRS) ranging from 0 (no pain at all) to 10 (the greatest pain imaginable). Swelling was classified into five grades: Grade I was defined as local swelling at the site of the bite; Grade II as redness and swelling up to the wrist or ankle joint; Grade III as redness and swelling up to the elbow joint or knee joint; Grade IV as redness and swelling extending to the entire limb; and Grade V as redness and swelling extending to the trunk or accompanied by systemic symptoms. Pain and swelling were assessed at presentation, at 6, 12, 24, 48, 72, and 96 h after presentation, and immediately before administration of antivenom. The primary endpoint was the length of hospital stay (in days), and secondary endpoints were adverse effects, pain, and grade of swelling.

Statistical analysis

Data are reported as the median and interquartile range for continuous variables, and as the number and percentage for categorical variables. Missing values were not computed. The Mann-Whitney U test was used to compare continuous variables between the two groups, and Fisher’s exact test was used to compare categorical variables. We used the log-rank test to investigate whether the length of hospital stay differed between the two groups. Because the proportional hazard assumption could not be assumed by visual inspection and Schoenfeld’s residuals test, we used generalized estimating equations with inverse probability of treatment weighting (IPTW) to examine the association between administration of mamushi antivenom serum and length of hospital stay after adjusting for background variables and administration of cepharanthine. The length of hospital stay was divided by the median and converted to a binary variable. Propensity scores were calculated using the variables, age, sex, Charlson Comorbidity Index, pain on arrival, and grade on arrival. The average treatment effect-weighted patients were entered into the generalized estimating equations to predict hospital stay of >4 days with 95% confidence intervals (CI). P-values of <0.05 were considered statistically significant. Statistical analyses were performed using SPSS software (IBM Corp. Released 2020. IBM SPSS Statistics for Windows, Version 27.0. Armonk, NY: IBM Corp).

## Results

**Figure 1:**
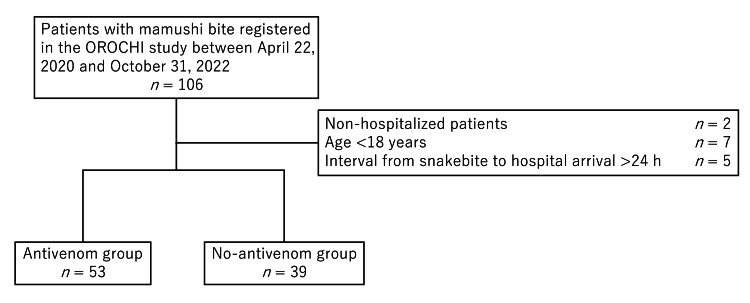
Patient flow diagram OROCHI, Observational Research Of Clinical course after mamusHI bite.

A total of 106 patients with mamushi bite were registered in the OROCHI study between April 2020 and October 2022 (Figure 1). Of these, 92 patients were eligible for the present analyses after excluding cases aged <18 years, non-hospitalized cases, and cases with an interval of >24 h between the snakebite and hospital arrival. There were 53 patients in the antivenom group and 39 in the no-antivenom group.

The patient characteristics are provided in Table 1. The median age of the patients was 72 years, 52% were male, the median time from snakebite to presentation was 65 min, and the median Charlson Comorbidity Index was 4; there were no differences in these characteristics between the two groups. Furthermore, there were no between-group differences regarding access to hospital, site of injury, prehospital first aid, basis of diagnosis, or Sequential Organ Failure Assessment score on arrival. However, the NRS score and grade of swelling on arrival were higher in the antivenom group than in the no-antivenom group.

**Table 1:** Patient characteristics IQR, interquartile range; NRS, numerical rating scale; SOFA, sequential organ failure assessment.

	Antivenom group (n = 53)	No-antivenom group (n = 39)	P
Age, years; median (IQR)	72 (62-83)	72 (65-76)	0.583
Male, n (%)	23 (43.4)	25 (64.1)	0.059
Charlson Comorbidity Index, median (IQR)	4 (3-5)	4 (3-5)	0.887
Interval between snakebite and hospital arrival (min), median (IQR)	65 (45-125)	65 (32-90)	0.293
Route to hospital, n (%)			0.404
Ambulance	18 (34.0)	16 (41.0)	
Walk-in	26 (49.1)	20 (51.3)	
Referral from another hospital	9 (17.0)	3 (7.70)	
Site of injury, n (%)			0.699
Hand	41 (77.4)	29 (74.4)	
Foot	10 (18.9)	8 (20.5)	
Forearm	1 (1.9)	0 (0.0)	
Lower leg	1 (1.9)	1 (2.6)	
Femoral	0 (0.0)	1 (2.6)	
Prehospitalization first aid, n (%)			
Suction	5 (9.6)	5 (13.2)	0.737
Binding	14 (26.9)	8 (21.1)	0.623
Incision	0 (0.0)	0 (0.0)	
Others	1 (1.9)	3 (7.9)	0.349
Basis of diagnosis, n (%)			
Bite mark	31 (58.5)	26 (66.7)	0.516
Symptoms	35 (66.0)	22 (56.4)	0.390
Sighting	41 (77.4)	28 (71.8)	0.629
Pain on arrival (NRS), median (IQR)	7 (4-7)	3 (2-7)	0.005
Grade of swelling on arrival, median (IQR)	2 (2-3)	2 (1-2)	0.002
SOFA score on arrival, median (IQR)	0 (0-0)	0 (0-1)	0.073

The hospital interventions are listed in Table 2. Incision of the bite site was performed in 10% of patients, antimicrobials were administered to 80%, and cepharanthine was administered to 80%, with no differences between the two groups. However, steroids were administered to a significantly greater percentage of patients in the antivenom group (49% vs. 15%, P < 0.001). In the antivenom group, 10% of patients received multiple doses of mamushi antivenom serum, the median time from snakebite to administration of antivenom was 300 min, an antivenom intradermal test was performed in 65% of patients, and antivenom desensitization was performed in 28% of patients.

**Table 2:** Hospital interventions IQR, interquartile range.

	Antivenom group (n = 53)	No-antivenom group (n = 39)	P
Incision, n (%)	5 (9.4)	4 (10.3)	1.000
Multiple doses of antivenom, n (%)	5 (10.0)	-	-
Time from snakebite to administration of antivenom (min), median (IQR)	300 (175-495)	-	-
Antivenom intradermal test, n (%)	33 (64.7)	-	-
Negative	17 (32.1)	-	-
Positive (mild hypersensitivity)	1 (1.9)	-	-
Positive (severe hypersensitivity)	0 (0.0)	-	-
Antivenom desensitization, n (%)	14 (27.5)	-	-
Administration of:			
Antimicrobials, n (%)	43 (81.1)	31 (79.5)	1.000
Cepharanthine, n (%)	43 (81.1)	31 (79.5)	1.000
Steroids, n (%)	26 (49.1)	6 (15.4)	<0.001
Relaxing incision, n (%)	1 (1.9)	0 (0.0)	1.000
Renal replacement therapy, n (%)	0 (0.0)	0 (0.0)	-

**Figure 2:**
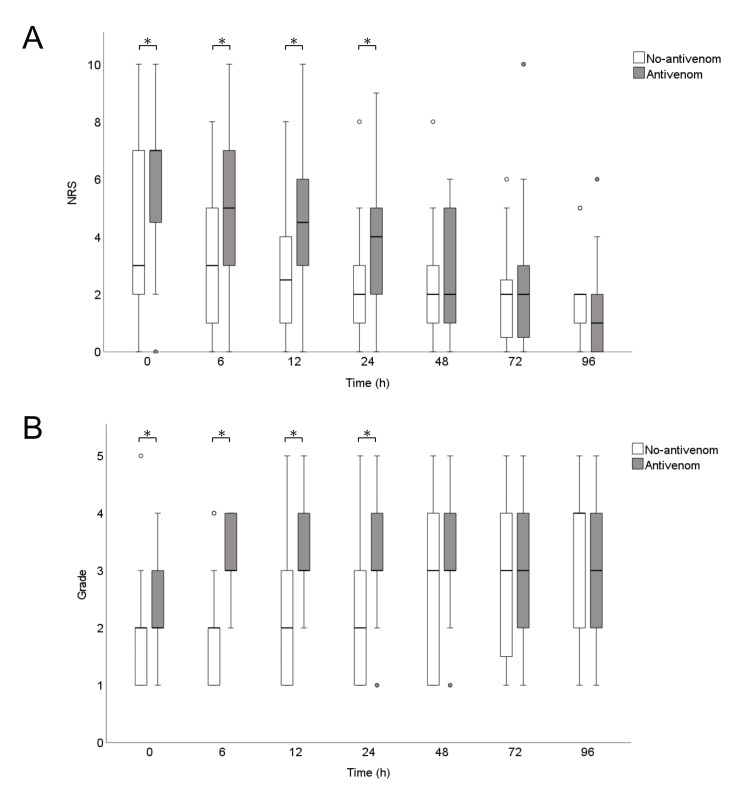
Box-and-whisker plots of the time-course of pain (A) and the grade of swelling (B) The white boxes represent the no-antivenom group, and the gray boxes represent the antivenom group. There were no significant differences in the numerical rating scale (NRS) or grade of swelling between the two groups after 48 h. The circles indicate outliers, defined as data points with values below the value of the first quartile minus one and a half times the interquartile range, or above the third quartile plus one and a half times the interquartile range. *P < 0.05. NRS, numerical rating scale.

The patient outcomes are provided in Table 3. Two patients in the antivenom group were transferred to a different institute because their condition improved, and the others were discharged alive. All patients in the no-antivenom group were discharged alive. The median (interquartile) length of hospital stay was not significantly different between the antivenom and no-antivenom groups (5 (3-6) days vs. 3 (1-8) days, P = 0.369). Pain and the grade of swelling were worse in the antivenom group than in the no-antivenom group up to 24 h after arrival, but there was no difference after 48 h (Figure 2). Adverse effects of mamushi antivenom serum were observed in four patients: anaphylaxis in one, nausea and vomiting in one, serum sickness in two, and rash in one.

**Table 3:** Patient outcomes IQR, interquartile range; NRS, numerical rating scale.

	Antivenom group (n = 53)	No-antivenom group (n = 39)	P
Adverse effects of antivenom	4 (7.5)	-	-
Anaphylaxis	1 (1.9)	-	-
Nausea and vomiting	1 (1.9)	-	-
Serum sickness	2 (3.80)	-	-
Rash	1 (1.9)	-	-
Pain (NRS), median (IQR)			
At antivenom administration	6 (5-8)	-	-
6 h after presentation	5 (3-7), n = 47	3 (1-5), n = 31	0.002
12 h	5 (2-6), n = 46	3 (1-4), n = 30	0.001
24 h	4 (2-5), n = 45	2 (1-3), n = 30	0.003
48 h	2 (1-5), n = 42	2 (1-3), n = 24	0.086
72 h	2 (0-3), n = 35	2 (1-3), n = 20	0.568
96 h	1 (0-2), n = 29	2 (1-2), n = 15	0.711
Worst pain	7 (5-8), n = 51	3 (2-7), n = 38	<0.001
Grade of swelling, median (IQR)			
At antivenom administration	3 (3-4)		
6 h after presentation	3 (3-4), n = 50	2 (1-2), n = 31	<0.001
12 h	3 (3-4), n = 47	2 (1-3), n = 30	<0.001
24 h	3 (3-4), n = 50	2 (1-3), n = 30	<0.001
48 h	3 (3-3), n = 44	3 (1-4), n = 24	0.145
72 h	3 (2-4), n = 36	3 (1-4), n = 19	0.964
96 h	3 (2-4), n = 31	4 (2-4), n = 15	0.410
At worst	4 (3-4), n = 53	2 (1-3), n = 39	<0.001
Outcomes, n (%)			0.506
Transfer due to improvement	2 (3.80)	0 (0.0)	
Survival discharge	51 (96.2)	39 (100.0)	
Length of hospital stay (days), median (IQR)	5 (3‒6)	3 (1‒8)	0.369

**Figure 3:**
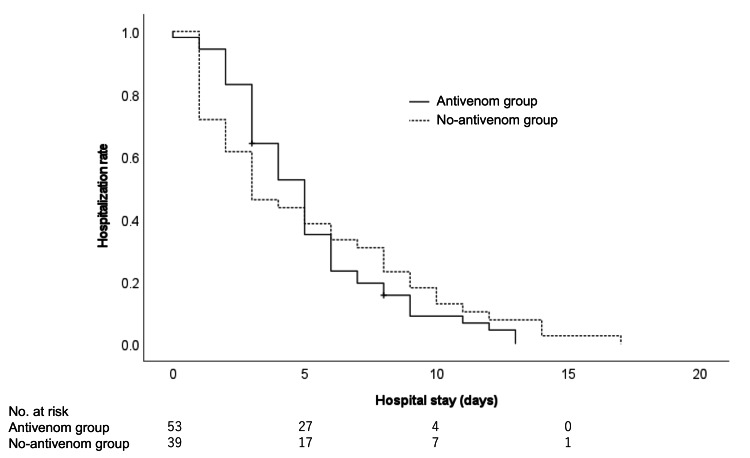
Kaplan-Meier curves of the length of hospital stay for patients in the antivenom and no-antivenom groups

The Kaplan-Meier curves comparing the length of hospital stay in both groups are shown in Figure 3. The log-rank test showed no significant difference between the two groups (P = 0.732). The patient characteristics after applying IPTW are provided in Table 4, and all variables showed better balance between the two groups. In the multivariable analysis, the adjusted odds ratio for a hospital stay of >4 days was 1.331 in patients treated with mamushi antivenom serum (95% CI 0.744‒2.015, P = 0.574; Table 5). In contrast, the adjusted odds ratio was 6.154 in patients treated with cepharanthine (95% CI = 1.442-26.258, P = 0.014), indicating a significant association between cepharanthine treatment and hospital stay of >4 days.

**Table 4:** Patient characteristics after IPTW The number of patients differs from the original numbers because inverse probability of treatment weighting was performed. IPTW, inverse probability of treatment weighting; SMD, standardized mean difference; IQR, interquartile range; NRS, numerical rating scale.

	Antivenom group (n = 86)	No-antivenom group (n = 77)	SMD
Age, years; median (IQR)	72 (63-83)	72 (67-80)	0.033
Male, n (%)	47 (54.5)	46 (60.1)	0.121
Charlson Comorbidity Index, median (IQR)	4 (3-5)	4 (3-5)	0.057
Pain on arrival (NRS), median (IQR)	5 (3-7)	6 (2-8)	0.004
Grade of swelling on arrival, median (IQR)	2 (1-2)	2 (1-2)	0.144

**Table 5:** Multivariable logistic regression analysis of factors associated with a hospital stay of >4 days Because of the inverse probability of treatment weighting, the final model only includes antivenom administration and cepharanthine administration. CI, confidence interval.

	Adjusted odds ratio (95% CI)	P
Antivenom administration	1.331 (0.491-3.609)	0.574
Cepharanthine administration	6.154 (1.442-26.258)	0.014

## Discussion

This prospective observational multicenter study was performed to investigate the effectiveness of mamushi antivenom serum in patients bitten by mamushi, but we found no effect of its administration on the length of hospital stay. Adverse effects of the antivenom were observed in four of the 53 patients, but some beneficial effects of the antivenom on pain and swelling were also observed. To the best of our knowledge, the effects of mamushi antivenom serum have only been reported in retrospective studies [2-7]. Therefore, ours is the highest quality study to date to examine the effectiveness of mamushi antivenom serum. Accordingly, our findings should be valuable for helping physicians decide whether to administer mamushi antivenom serum in clinical practice.

Because of the very low mortality rate of mamushi bites [1], we used the length of hospital stay as the primary endpoint in this study. One retrospective questionnaire-based study in tertiary care centers examined the effects of mamushi antivenom serum and cepharanthine [2]. The authors reported shorter hospital stay in patients with Grade III/V swelling in the mamushi antivenom serum alone group than in the cepharanthine alone group, but the authors did not make comparisons between the antivenom and no-antivenom groups. Several single-center retrospective studies have also been published [3-7]. Some, but not all, of those studies reported that mamushi antivenom serum reduced the length of hospital stay. Thus, the evidence does not fully prove whether administration of mamushi antivenom serum has a significant effect on the length of hospital stay.

By comparison, prior reports described consistent evidence regarding the effectiveness of mamushi antivenom serum on reducing swelling and systemic symptoms, although only single-center, retrospective studies are available. A study of 78 patients who received mamushi antivenom serum within 24 h of presentation versus 20 patients who did not receive antivenom showed that fewer patients experienced Grade V swelling in the antivenom group [5]. In a study of 114 patients, systemic symptoms occurred in 8.4% of patients who received mamushi antivenom serum within 3 h of being bitten, compared with 53.8% of patients who received cepharanthine alone [4]. In another study of 57 patients who received mamushi antivenom serum, the authors performed receiver operating characteristic curve analysis to predict deterioration to Grade V based on the interval between the snakebite and antivenom administration [9]. The authors reported that the area under the curve was 0.816, and the sensitivity and specificity were 80% and 81%, respectively, for a cutoff value of 14 h [9]. In the present study, swelling and pain were significantly worse up to 24 h after the patient’s arrival at the hospital, but there was no difference after 48 h, suggesting that antivenom is effective for reducing swelling and pain.

Mamushi antivenom serum is prepared from horse serum, and adverse effects can be a problem. In the present study, adverse effects were observed in 8% of patients treated with the antivenom. In previous reports, the incidence of adverse effects in patients treated with mamushi antivenom serum ranged from 2% to 15% [1-3,5-7,9-10], consistent with the present results. To the best of our knowledge, there are no reports of deaths due to mamushi antivenom serum administration. However, anaphylaxis was observed in one patient in this study. Therefore, it is imperative that mamushi antivenom serum be administered in a clinical environment where it is possible to respond immediately to anaphylaxis.

In the present study, cepharanthine administration was significantly associated with a hospital stay of >4 days. Reports of the association between cepharanthine and this outcome are limited, but a retrospective observational multicenter study reported that severely ill patients treated with cepharanthine alone tended to be hospitalized for longer than patients who did not receive either cepharanthine or mamushi antivenom serum [2]. Another single-center retrospective study revealed that patients treated with the cepharanthine had a significantly longer hospital stay than patients treated without cepharanthine, but the former group tended to have a higher grade of swelling at presentation [6]. In the 1950s, cepharanthine was reported to be effective against mamushi bites and it has been used in the treatment of mamushi bites for a long time because it has few side effects. However, cepharanthine is possibly harmful in patients with mamushi bites, and further research is needed.

There are several limitations of the present study. First, this was an observational study and there may be unmeasured or unknown confounders. Second, the relatively small number of cases may have resulted in insufficient statistical power to detect significant differences in the study outcomes. However, mamushi bites are relatively rare and a national registry would be needed to collect more cases. Third, the decision to hospitalize the patient, the decision to discharge the patient from hospital, and the diagnosis of the side effects of the mamushi antivenom serum were made by the attending physician, and this may introduce some bias. Finally, the observation period was only until discharge from the hospital, and some delayed adverse effects may have been missed.

## Conclusions

We analyzed data from the OROCHI study, a multicenter prospective observational study, to examine the effect of mamushi antivenom serum on the length of hospital stay after mamushi bite. Although the study could not demonstrate the effect of mamushi antivenom serum on reducing the length of hospital stay, some beneficial effects on swelling and pain were observed. The findings from this study will be useful when treating patients bitten by mamushi.

## References

[1] Yasunaga H, Horiguchi H, Kuwabara K, Hashimoto H, Matsuda S (2011). Venomous snake bites in Japan. Am J Trop Med Hyg.

[2] Hifumi T, Yamamoto A, Morokuma K (2013). Clinical efficacy of antivenom and cepharanthine for the treatment of mamushi (Gloydius blomhoffii) bites in tertiary care centers in Japan. Jpn J Infect Dis.

[3] Kochi K, Okita M, Ito T, Shigemoto S (1995). A study of 50 cases of mamushi bite. J Jpn Pract Surg Soc.

[4] Makino M, Yurugi E, Abe J (1988). A study of 114 cases of viper bite: with special reference to the administration of antivenin. J Jpn Pract Surg Soc.

[5] Furuuchi K, Dote H, Atsumi T, Suganuma K, Hayakawa T (2022). Analysis of 108 mamushi pit viper bite cases. (Article in Japanese). J Jpn Soc Emerg Med.

[6] Yoshimine S, Seyama A, Suga A (2019). Clinical study of 67 cases of Japanese mamushi viper (Gloydius blomhoffii) bite. (Article in Japanese). J Rural Med.

[7] Noda K, Akiyama N, Ii K (2017). The effects of early treatment with anti-venom on length of hospital stay: analysis of 46 cases of mamushi bites. (Article in Japanese). Chudoku Kenkyu.

[8] Hifumi T, Yamamoto A, Morokuma K (2011). Surveillance of the clinical use of mamushi (Gloydius blomhoffii) antivenom in tertiary care centers in Japan. Jpn J Infect Dis.

[9] Okamoto O, Sato T, Todoroki A (2020). Statistical analysis of anti-mamushi venom serum injection time and clinical course. Acute Med Surg.

[10] Chiba T, Koga H, Kimura N (2018). Clinical condition and management of 114 mamushi (Gloydius blomhoffii) bites in a general hospital in Japan. Intern Med.

